# Periacetabular osteotomy with or without femoral osteotomy for the treatment of hip subluxation in children and young adults with cerebral palsy

**DOI:** 10.1186/s12891-022-05754-3

**Published:** 2022-08-25

**Authors:** Kangming Chen, Jinyan Wu, Chao Shen, Junfeng Zhu, Xiaodong Chen, Jun Xia

**Affiliations:** 1 Department of Orthopaedics, Huashan Hospital, Fudan University, No.12 Middle Wulumuqi Road, Jing’an District, 200040 Shanghai, China; 2 Department of Orthopaedics, Xinhua Hospital affiliated to Shanghai Jiao Tong University, School of medicine, No.1665, Kongjiang Road, Yangpu District, Shanghai, 200092 People’s Republic of China

**Keywords:** Cerebral palsy, Hip subluxation, Periacetabular osteotomy, Femoral osteotomy

## Abstract

**Background:**

This study is aimed to investigate retrospectively the radiographic and clinical outcomes in children and young adults with cerebral palsy (CP) undergoing periacetabular osteotomy (PAO) with or without femoral osteotomy (FO) for hip subluxation.

**Methods:**

A consecutive cohort of twenty-one patients (23 hips) with symptomatic CP hip subluxation were treated with PAO with or without FO and reviewed retrospectively. Two patients (2 hips) were excluded due to insufficient follow-up and lost to follow-up, respectively. The Reimers migration percentage, lateral center-edge angle (LCEA), Sharp angle, neck-shaft angle (NSA), femoral anteversion (FNA), Gross Motor Function Classification System (GMFCS) and hip pain were assessed.

**Results:**

Twenty-one hips (19 patients) with CP treated with PAO with or without FO were included. Five hips received PAO. Sixteen hips underwent PAO with FO. Mean age at surgery was 19 ± 6 and 15 ± 4 years for PAO and PAO plus FO, respectively. Mean follow-up was 44.0 ± 28.3 months for PAO and 41.5 ± 17.2 months for PAO + FO. All hips were painful before surgery and painless at final visits. The GMFCS improved by one level in 10 of 19 patients. There was significant increase in LCEA (*p* < 0.001) and decrease in the Reimer’s MP (*p* < 0.001), NSA (*p* < 0.001) and Tonnis angle(*p* < 0.001) postoperatively. Resubluxation occurred in 7 hips (30%) due to insufficient correction and loosening of fixation. Nervus cutaneus femoris lateralis was impaired in 4 patients after surgery. There was no avascular necrosis of the femoral head, resubluxation or infection.

**Conclusion:**

PAO with or without FO can be effective for children and young adults with concomitant hip subluxation and CP.

## Introduction

Hip subluxation is a common disorder in children with cerebral palsy (CP) [1]. Muscular imbalance is the primary cause, which leads to unequal loading, followed by migration of the head and unbalanced growth. Migration presents as subluxation and dislocation. The incidence of hip subluxation ranges from 2 to 75% [2–4]; variation in the incidence may be attributed to different definitions, measuring methods and patient age at measurement. The prevalence of dislocation ranges from 10 to 15% [5, 6]; subluxation rate has been estimated to be between 25 and 60% [7, 8]. Hip subluxation may cause pain, limit hip function and worsen quality of life. Hip pain has been reported to present in 33 to 70% of unstable hips with CP [9, 10]. It might be the main reason for surgical hip reconstruction [11].

Isolated femoral varus derotation osteotomy (VDRO) has been reported to create a stable and concentrically reduced hip joint in young children with CP [12, 13]. However, resubluxation with high risk would ensue after isolated VDRO, since acetabular remodeling is limited [14]. In addition, VDRO can shorten the distance between the origin and insertion of gluteus medius, and therefore weaken the muscle strength, at least temporarily. Therefore, treatment with concurrent acetabular osteotomies and femoral osteotomy are considered for over 70% of patients with femoral head migration [15], especially for those over 9 years old.

Several pelvic osteotomies, including Salter osteotomy, Chiari osteotomy, Pemberton osteotomy, triple pelvic osteotomy and Dega Osteotomy, have been advocated for dislocated hips [11, 16–21]. Chiari is an augmentation, with Pemberton and Dega being halfway reorientations while Salter and triple osteotomies are true reorientations through which the acetabular growth plate can be preserved. In addition, spica casts are recommended for most cases. In the literature, pelvic procedures in CP children are followed by various complications, such as resubluxation, hardware infection, as well as avascular necrosis of femoral head [3, 22].

Ganz et al. [23] developed periacetabular osteotomy (PAO), through which surgeons are able to perform multiplanar correction to the deformities via a single incision without disrupting posterior column integrity, true pelvis shape, or vasculature to acetabulum. A realigned acetabulum with stability means less restrictions to bearing weight after surgery. PAO has become our primary acetabular realignment osteotomy in treating developmental dysplasia of the hip. Complications of PAO should not be ignored, but detailed technical execution flattens the learning curve with increasing caseload [24–26].

Although the acetabular morphology in CP patients is different from that in DDH patients [27] and softer bone makes osteotomies and correction maneuvers more difficult, the advantages of PAO led us to think that it might be a possible solution to hip subluxation in CP patients.

The primary goal of this study was to assess the clinical and radiological outcome of PAO with or without FO on subluxated hips in CP patients. Our secondary goal was to evaluate complications that followed these procedures.

## Methods

**Fig. 1:**
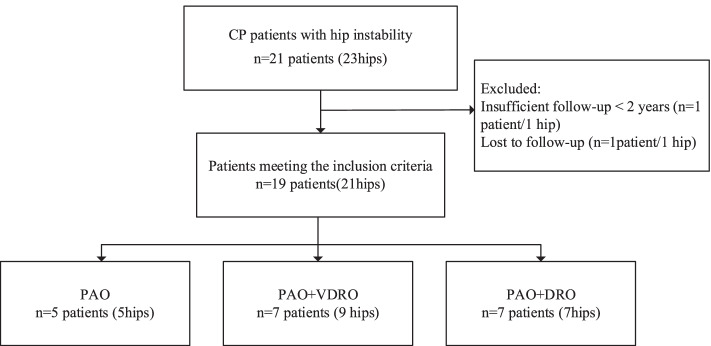
Flow chart of inclusion and exclusion. PAO, periacetabular osteotomy. VDRO, varus derotation osteotomy. DRO, derotation osteotomy

After receiving the approval from the IRB at our institute, we retrospectively reviewed the surgical treatment of 21 consecutive children and young adult patients with symptomatic hip dysplasia associated with spastic CP. Inclusion criteria were: a diagnosis of cerebral palsy, surgical treatment of hip subluxation (migration percentage, MP, > 30%) by either unilateral or bilateral PAO with or without FO, clinical and radiographic follow-up of at least 2 years from the time of the index surgery. Exclusion criteria were: severe deformity of the femoral head after long-term dislocation; subtle displacement of the hip joint (MP ≤ 30%); insufficient follow-up duration (< 2 years). Two patients (2 hips) were excluded. One patient had done well initially at 3, 12 and 18 months but subsequently lost to follow-up. Another patient had follow-up time less than 2 years. This left 19 patients (21 hips) to be included for analysis of surgical technique and assessment of early outcome (Fig. 1).

### Surgical techniques

All procedures were performed under general anesthesia, with the patient lying supine. The first step was an open soft tissue release, including tenotomies of adductor longus, adductor brevis and gracilis, on all patients. The iliopsoas tendon was lengthened (released or pie-crusted) for hip flexion contracture of > 20°, which was done during PAO via the same incision. But now, we perform release or pie-crusting once we feel that the iliopsoas is tight during PAO. The affected lower limb was internally rotated and then abducted to evaluate the congruence between the acetabulum and femoral head with fluoroscopy. The neck-shaft angle (NSA) was reassessed by fluoroscopy simultaneously. Subtrochanteric femoral derotation osteotomy (DRO) was performed prior to PAO in patients with femoral neck anteversion (FNA) over 40° (NSA less than 140°) and fixed with 4.5-mm LC-DCP (Synthes, USA). If the NSA exceeded 140° and the femoral neck anteversion was greater than 40°, intertrochanteric femoral varus derotation osteotomy (VDRO) would be performed to restore the femoral anteversion to 15° ~ 20° and NSA to 115° ~ 125°. A 5.0-mm proximal femoral locking plate (Synthes, USA) was used for fixation. Femoral shortening of 1.0 to 1.5 cm was performed if FO was necessary.

PAO was performed according to the method described by Ganz et al. [23] through a modified Smith-Petersen approach by a senior surgeon on all patients. The periacetabular osteotomies must be completed with instruments instead of fracturing the final bridges. Otherwise, insufficient correction will ensue. A reasonable amount of bone was removed routinely from proximal pubis osteotomy end. This will facilitate manipulation of the fragment. The acetabular fragment was provisionally fixed with 2.5-mm K-wires. The acetabular inclination, rotation center, Shenton line and femoral head coverage were intraoperatively assessed with fluoroscopy. Finally, the acetabular fragment was fixed with three or four 3.5-mm cortical screws (Synthes, USA). Hip capsule was opened routinely to evaluate the cartilage and labrum. The elongated ligamentum teres was excised. Osteochondroplasty was done to restore the offset of femoral neck. Labrum was repaired if torn. The capsule plication was done in highly subluxated hip. The anterior superior iliac spine fragment was fixed to its original site with a 3.5-mm cortical screws.

Drainage was used in every case. No spica cast was applied in any of the patients. The patients were encouraged to get out of bed after removal of their drainage tubes and to walk with two crutches without weight-bearing for six to 8 weeks after surgery. Full weight-bearing was allowed if radiographs showed evidence of healing in osteotomy site. Rehabilitation of gluteal musculature was recommended after surgery to prevent contracture.

Patients were followed 6 weeks, 3 months, 6 months, 12 months and then annually after surgery. Medical records were reviewed for the presence of pain. All patients’ pre-operative, initial and the latest post-operative anteroposterior pelvic radiographs, as well as any sign or symptom, were recorded. The radiographic indices including Reimers MP, LCEA, Tonnis angle, NSA were measured. CT scan with volume rendering [21] was routinely taken to assess the acetabular deficiency and femoral neck anteversion pre-operatively. The Gross Motor Function Classification System (GMFCS) for CP was assessed pre- and postoperatively. Pain was evaluated with visual analogue scale (VAS) preoperatively and at the final follow-up.

### Statistical analysis

Statistical analyses were performed with R 4.2.0 (R Foundation for Statistical Computing). Shapiro-Wilk test was used to test normality. Generalized estimating equation (GEE) was used to accommodate correlations among three points of time while adjusting for sex and age at surgery. Post hoc procedures were followed for within-group and between-group analyses. Independent t test and Mann Whitney U test were used to compare pre-operative and post-operative alignments in hip whose LCEA were less than 20 degrees. Significance level was set at 0.05.

## Results

From October 2013 to December 2018, a total of 21 hips in 19 patients with cerebral palsy who underwent a PAO alone or combined with FO were included in this study (Table 1). There were 11 males and 8 females. The mean age was 16.2 years (range, 7 to 28 years) at the index surgery. Fifteen patients had diplegia, three having quadriplegia and one having hemiplegia. Three patients with scoliosis had posterior instrumentation prior to the index surgery. One patient had signs of pelvic osteotomy in early childhood. One patient had undergone femoral osteotomy. Four patients had had soft tissue release. One patient had prior selective posterior rhizotomy (SPR). 5 patients received PAO. Fourteen patients underwent PAO combined with FO (Table 2). The mean follow-up duration was 41.2 months (range, 24 to 86 months).

**Table 1:** Summary of patients (hips)

patient	hip	Gender	age at index surgery (years)	side	hemi/di/ or quadriplegia	prior hip surgery	Procedures	Pre-op GMFCS	Post-op GMFCS	Follow-up duration (months)
1	1	M	13	R	quadriplegia	STR	PAO + DRO	II	I	57
2	2	M	21	R	diplegia	STR	PAO + VDRO	II	II	53
3	3	M	24	R	diplegia		PAO + VDRO	III	III	41
4	4	M	15	R	hemiplegia	SPR	PAO + VDRO	II	I	31
5	5	M	14	R	diplegia	STR	PAO	III	III	27
6	6	M	15	L	diplegia		PAO	II	I	27
7	7	M	17	R	diplegia		PAO + DRO	III	III	28
8	8	M	20	R	diplegia	pelvic osteotomy	PAO + DRO	III	II	24
9	9	F	17	R	diplegia		PAO + DRO	II	I	25
10	10	M	15	R	hemiplegia		PAO + DRO	III	II	24
11	11	F	28	L	diplegia	Spine fusion	PAO	IV	III	36
12	12	F	7	R	diplegia		PAO + VDRO	II	I	35
13	13	F	12	R	diplegia		PAO + VDRO	III	III	50
14	14	F	14	L	diplegia		PAO + DRO	II	I	65
/	15	/	/	R	/	femoral osteotomy	PAO	/	/	/
15	16	M	15	R	diplegia		PAO + VDRO	III	III	69
16	17	F	19	R	quadriplegia	Spine fusion	PAO	III	III	86
17	18	M	14	R	diplegia		PAO + VDRO	IV	IV	68
18	19	F	14	L	diplegia	STR	PAO + VDRO	II	I	28
/	20	/	/	R	/		PAO + VDRO	/	/	/
19	21	F	14	R	quadriplegia	Spine fusion	PAO + DRO	V	IV	25

*PAO* indicates periacetabular osteotomy, *SPR* Selective posterior rhizotomy, *STR* Soft tissue release, *DRO* subtrochanteric femoral de-rotation osteotomy, *VDRO* Intertrochanteric femoral varus de-rotation osteotomy, *GMFCS* Gross motor function classification system

**Table 2:** Demographics of patients in two groups

	PAO	**PAO + FO**	*p* value
Patients	5	14	–
Surgeries	5	16	–
Male/Female	5/0	6/8	0.045^*^
Age (years)	19 ± 6	15 ± 4	0.356^***^
Follow-up duration	44 ± 28.3	41.5 ± 17.2	0.960^**^

*Fisher’s exact test

**Mann-Whitney U test

***independent two-sample t test

*PAO* Periacetabular osteotomy, *FO* Femoral osteotomy

All hips were painful preoperatively and the patients could not walk or sit for long periods. The VAS value improved significantly at final follow-up as compared with pre-operation(*p* < 0.001). All patients showed improved personal hygiene because of abduction improvement and pain relief. Sitting and/or walking ability were also improved. The GMFCS improved by one level in 10 out of 19 patients at final follow-up.

### Radiological measurements

**Fig. 2:**
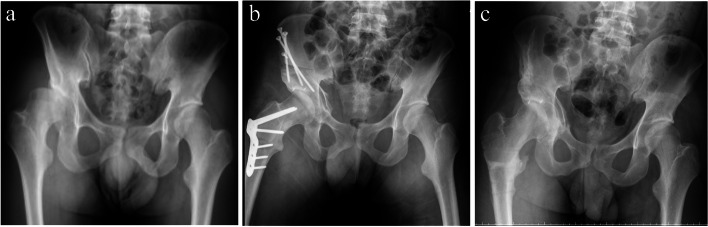
A 21-year-old male, diplegia. Pre-op GMFCS was II. Post-op GMFCS was II. **a** Pelvic radiography before surgery; **b** immediately after surgery of PAO + VDRO as well as labrum repair; **c** two years after surgery

**Fig. 3:**
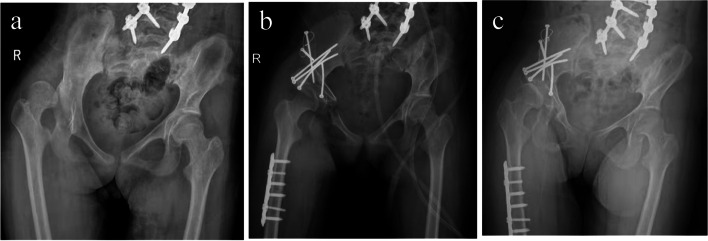
A 14-year-old female, quadriplegia. Pre-op GFMCS was V while post-op GFMCS was VI. Anteroposterior pelvic radiograph **a** before surgery; **b** immediately after PAO + DRO; **c** one year after surgery

The acetabular deficiency was located posterolaterally in 14 hips, laterally in 6 hips, and anterolaterally in 1 hip. Descriptive statistics for all alignments and VAS were tabulated in Table 3. There was significant increase in LCEA and decreases in the Reimers MP, NSA and Tonnis angle at the final follow-up in patients undergoing PAO with FO (*p* < 0.001). There was significant increase in LCEA and adecrease in the Reimers MP, Tonnis angle, as well as VAS, at initial post-operation and final follow-up in patients undergoing PAO with or without FO (*p* < 0.001) (Table 4) (Figs. 2 and 3). There was no significant difference in LCEA at initial or final post-operative measurement between PAO group and PAO plus FO group. There were no significant differences between the two groups (PAO alone and PAO with FO) regarding LCEA, Tonnis angle, Reimers MP and NSA at final follow-up (*p* > 0.05) (Table 5). However, the hips receiving PAO with FO had higher FNA, NSA (*p* < 0.005) (Table 5). Although there was significant difference in MP, and Tonnis angle between the initial post-operative measures and those at the final follow-up (*p* = 0.001 and < 0.001, respectively), this is not clinically relevant. Nevertheless, there was no significant difference in LCEA between the initial and final post-operative measures (*p* = 0.051).

**Table 3:** Descriptive statistics for radiographic indices and VAS before/after PAO with/without FO

	PAO			PAO + FO		
	pre-op	initial post-op	post-op	pre-op	initial post-op	post-op
NSA (°)	137 ± 5.1	137 ± 5.1	137 ± 5.1	143.7 ± 6.2	133.0 ± 7.6	132.4 ± 8.0
LCEA (°)	−16.5 ± 21.6	25.4 ± 5.7	24 ± 7.7	−38.7 ± 21.6	23.7 ± 4.6	20.7 ± 10.5
MP (%)	61.1 ± 17.4	18.0 ± 10.3	19.8 ± 11.2	81.4 ± 16.3	17.4 ± 10.2	19.5 ± 12.2
Tonnis Angle (°)	27 ± 10.4	7.1 ± 7.5	8 ± 8.2	37.5 ± 9.8	11.6 ± 7.5	13 ± 8.8
FNA (°)	26.7 ± 9.9	–	26.7 ± 9.9	44.1 ± 11.6	–	21.4 ± 1.5
VAS	5 ± 1	–	0.2 ± 0.45	5.4 ± 0.9	–	0.3 ± 0.4

*NSA* Neck shaft angle, *LCEA* Lateral center-edge angle, *MP* Reimer migration percentage, *FNA* Femoral neck anteversion, *PAO* Periacetabular osteotomy, *FO* Femoral osteotomy, *VAS* visual analogue scale, *pre-op* Pre-operative; post-op, post-operative. Post-op alone refers to post-operative measurements at the final follow-up

**Table 4:** Within-group differences among pre-op, initial post-op and post-op at the last follow-up measures after adjusting for age at surgery and sex

	Pre-op minus initial post-op				post-op minus initial post-op				post-op minus pre-op			
	PAO	*p* value	PAO + FO	*p* value	PAO	*p* value	PAO + FO	*p* value	PAO	*p* value	PAO + FO	*p* value
NSA (°)	–	–	10.67	< 0.001	–	–	− 0.558	0.024	–	–	−11.233	< 0.001
LCEA (°)	− 42.0	< 0.001	−62.4	< 0.001	−1.5	0.13	− 3.0	0.051	40.5	< 0.001	59.4	< 0.001
MP (%)	43.1	< 0.001	64.0	< 0.001	1.8	0.002	2.1	0.001	−41.4	< 0.001	− 61.9	< 0.001
Tonnis Angle (°)	19.9	< 0.001	25.9	< 0.001	0.9	0.023	1.5	< 0.001	−19	< 0.001	−24.5	< 0.001
FNA(°)	–	–	–	–	–	–	–	–	–	–	−22.7	< 0.001
VAS	–	–	–	–	–	–	–	–	−4.8	< 0.001	−5.1	< 0.001

Using generalized estimating equations (GEE), we introduced the terms of age at surgery and sex to control for their confounding effects, as well as the correlations among three points of time. Values under PAO and PAO + FO are the regression coefficients for dummy variables, which are the adjusted difference between each pair. Post-op alone refers to post-operative measurements at the final follow-up

**Table 5:** Between-group differences of measures at three points of time with adjustment for age at surgery and sex

	Pre-op		Initial post-op		Post-op	
	PAO + FO minus PAO	*p* value	PAO + FO minus PAO	*p* value	PAO + FO minus PAO	*p* value
NSA	5.77	0.014	−4.88	0.076	−5.46	0.055
LCEA	−24.15	0.006	−3.69	0.23	−5.22	0.21
MP (%)	20.3	0.02	−0.6	0.92	−0.2	0.97
Tonnis Angle	12.61	0.003	6.58	0.006	7.16	0.061
FNA	−5.0	0.311	–	–	−27.7	< 0.001
VAS	0.1	0.649	–	–	−0.3	0.629

Using GEE, we controlled for sex and age at surgery, as well as correlations among measures at three points of time. Values under ‘PAO + FO minus PAO’ are the regression coefficients for dummy variables, which are the adjusted difference between each pair of PAO plus FO and PAO alone. Post-op alone refers to post-operative measurements at the final follow-up

### Complications

Re-subluxation occurred in 7 hips (30%) in 6 patients (3 males, 3 females). Five patients were diplegic; one hemiplegic. Two patients were in GMFCS II; three in IV; one in V. Two hips received isolated PAO. Three hips underwent PAO combined with DRO. Two hips had PAO combined with VDRO. The mean pre-operative Reimers’ MP, LCEA and Tonnis angle in these hips improved from 75.6%, − 31.7 ° and 38.9 to 29.8%, 11.1 ° and 17.3 at final follow up, respectively (*p* < 0.0001, =0.0004 and 0.0005, respectively), yet the LCEA did not reach 20 ° (the mean LCEA was 11.1 °; range, − 11.4 ° to 18.4 °) after surgery. Hence, these hips still fell into the category of re-subluxation. The differences in changes of LCEA, MP and Tonnis angle between the initial and last post-operative measures were significant in the LCEA ≥20° group compared with the LCEA< 20° group (*p* = 0.005, < 0.001 and 0.040 respectively.). There was no significant difference between the LCEA ≥20° group and the LCEA< 20° group in mean MP, LCEA and Tonnis angle pre-operatively. Differences were significant between the LCEA ≥20° group and the LCEA< 20° group in mean MP, LCEA and Tonnis angle at initial post-operative measures (*p* < 0.001, < 0.001, =0.0378, respectively). Despite the suboptimal postoperative hip alignment, there was no pain in these re-subluxated hips at the last visit. There was no significant difference between the LCEA ≥20° group and the LCEA< 20° group in mean VAS at latest follow up (*p* > 0.05).

Lateral femoral cutaneous nerve impairment was found in 4 patients. Numbness disappeared 6 months after surgery in 2 patients. The numbness region diminished in 2 patients 1 year after surgery. No patient developed AVN, complete re-dislocation, surgical site infection, sciatic nerve impairment or pressure sore at latest follow-up.

## Discussion

Untreated hip subluxation in patients with CP often results in pain, limitations in walking and sitting, and increased difficulty in nursing care within its natural course. Treating hip subluxation for patients with CP is challenging and the choice of treatments remains an open issue.

PAO has been used for treatment of DDH for more than 30 years. Satisfactory results have been reported in a variety of studies [28–30]. To our knowledge, this is the first study on the application of PAO on unstable hips in young adults with CP. Our results demonstrated that PAO with or without FO (DRO or VDRO) could relieve of the pain and was fairly effective in correcting the acetabular and proximal femoral deformity in CP patients. Hip pain was the main reason for patients’ seeking surgical interventions in this cohort. No hips presented pain at a mean 41.2-month follow-up. The GMFCS improved by one level in 10 out of 19 patients.

VDRO combined with a variety of pelvic osteotomies has been reported to be successful in treating severe subluxated or dislocated hips in CP children. Dega osteotomy and Pemberton osteotomy were the mainstream acetabuloplasties used extensively [17, 31]. These procedures were indicated for children with open triradiate cartilage and rarely reported in young adults. In addition, the acetabular deformity in CP patient is different from that in DDH. The acetabular deficiency is variable in CP patient, mainly located in posterolateral and lateral acetabulum [11, 27]. Our results demonstrated that 66.7% of acetabular deficiency was posterolateral, 28.6% lateral, which is in accordance with the literature [11, 27]. We believe PAO can correct deformity by manipulating the acetabular fragment to address the deficiency of the acetabulum.

No statistically significant difference was observed between two groups (isolated PAO and PAO combined with FO) with regard to LCEA, Tonnis angle, and Reimers MP post-operatively. However, we identified higher NSA, FNA in patients underwent PAO combined with FO preoperatively. This indicated that PAO and PAO combined with FO achieved equal outcomes in correcting hip subluxation at least radiographically.

Complications and reoperations are relatively common in acetabuloplasty procedures for patient with CP [3, 21, 22, 32–34]. McNerney et al. reported an 8% incidence of AVN in their study [3], with an even higher incidence in patients with open triradiate cartilage (68.7%) [33]. They proposed that disruption of the blood supply to the femoral head by lengthening soft tissue in the groin, and excessive pressure on the femoral head might contribute to AVN. In addition, Reimers MP of over 65% is associated with a higher rate of postoperative AVN [32].

No AVN occurred in this study. The mean Reimers MP was 76.6% preoperatively and improved to 18.6% at the final follow-up. It seems that higher Reimers MP and larger operative corrections did not necessarily trigger AVN after PAO combined with or without FO. Moreover, PAO is performed from inside of the pelvis where there are no substantial blood vessels supplying the femoral head. In contrast, other acetabular surgeries are mostly performed at the outside of the pelvis with the majority of femoral and acetabular vessels [35]. The mean age at surgery was 16.2 years in this cohort, which was greater than that in the previous reports with combined osteotomies [3, 32–34]. Mean age at surgery was 8.82 years in the cohort of Phillips (AVN,27%) [32], 9 years in Koch’s (AVN,68.7%) [33], 7.4 years in Khalife’s (AVN,37%) [34]. This probably meant that the younger the patient, the more prone to AVN.

Although significant improvement was obtained in LCEA and Reimers MP in these hips after surgery (Tables 6 and 7), LCEA did not reach 20 ° in 7 hips (30%). These hips should be categorized as resubluxation if the cutoff value for LCEA was set at < 20 °. However, only three hips (14%) can be categorized as resubluxation if the MP of < 30% was the cutoff value. Our results (mean pre-operative MP was 75.6%) are in accordance with previous reports that a preoperative migration percentage of > 50% was associated with poor results [36, 37]. We also found that the difference in the change of MP, LCEA and Tonnis angle from initial post-operative measures to the final follow-up was significant in the LCEA ≥20° group compared with the LCEA< 20° group (The *p* values were < 0.0001, 0.0053 and 0.0396, respectively.). There was no difference between the LCEA ≥20° group and the LCEA< 20° group in mean MP, LCEA and Tonnis angle before surgery. This means that there was some loss in acetabular fragment fixation. The poor bone quality would contribute to the fixation loss. Currently, we use 4 to 6 screws to reinforce the fixation of acetabular fragment in our daily clinical practice. Nevertheless, the acetabular plasticity observed in children after acetabuloplasty [16] would not happen in patient with closed triradiate cartilage, we believe that re-subluxation was also probably the result of suboptimal correction in this group of patients with a mean age of 16.2 years. Incomplete disconnection of the acetabular fragment and spastic muscles as well as soft bone may be the factors contributing to insufficient correction. (There was significant difference between the LCEA ≥20° group and the LCEA< 20° group in mean MP, LCEA and Tonnis angle at initial post-operation (*p* < 0.001, < 0.001, =0.038, respectively). The iliopsoas tendon was released for hip flexion contracture of > 20°. Now, we will perform release or pie-crusting once we feel that the iliopsoas is tight during PAO. Along with removing some bone from proximal pubis osteotomy end, these will facilitate elevating the teardrop without resistance from the tight iliopsoas. Then, a horizontal acetabular roof can be obtained. Of course, complete disconnection of acetabular fragment is the prerequisite. Applying botulinus toxin before surgery would probably be helpful for manipulating the acetabular fragment to a proper position and will certainly facilitate rehabilitation.

**Table 6:** Descriptive statistics for radiographic indices in patients with re-subluxation

*N* = 7 hips	Pre-operation	Post-operation	*p* value
Reimers MP (%)	75.6 ± 11.9	29.8 ± 9.1	< 0.001^*^
LCEA (°)	−31.7 ± 13	11.1 ± 10.6	< 0.001^*^
Tonnis Angle (°)	38.9 ± 9.9	17.3 ± 11.3	< 0.001^*^
NSA (°)	143.5 ± 6.7	135.2 ± 8.5	0.1814^**^
VAS	5.0 ± 0.6	0.3 ± 0.5	< 0.001^*^

*Independent two-sample t test

**Mann Whitney U test

Re-subluxation is denoted by post-operative LCEA of under 20 degrees at the last visit

*NSA* Neck shaft angle, *LCEA* Lateral center-edge angle, *MP* Reimer migration percentage

**Table 7:** Comparison between hips with and without re-subluxation

Alignments	Difference in the initial and the last post-op changes^a^		Difference at the initial post-operative measure^b^	
	Difference	*p* value	Difference	*p* value
LCEA	−7.6 ± 2.7	0.005	−8.0 ± 1.6	< 0.001
Reimers MP (%)	4.0 ± 0.8	< 0.001	12.3 ± 3.2	< 0.001
Tonnis Angle	1.8 ± 0.9	0.040	6.0 ± 2.9	0.038

^a^The difference refers to the difference in changes of a certain alignment, (((last post-operative PAO + FO)-(initial post-operative PAO + FO))-((last post-operative PAO)-(initial post-operative PAO))), with adjustment for age and sex

^b^The difference refers to the difference in alignments between PAO and PAO + FO at the initial post-operative measurement

*LCEA* Lateral centre-edge angle, *Reimers MP* Reimers migration percentage

This study has its limitations. This is a retrospective study with a small sample size and no control group. Actually, the CP patients who came to our adult reconstruction clinic were young adults, which is extremely challenging for orthopaedic surgeons. Pelvic osteotomy such as Salter osteotomy, Chiari osteotomy, Dega osteotomy and Pemberton osteotomy may not be appropriate for this group of patients. Hence, the control group was not possible. We are not able to predict the long-term survival rate because the follow-up periods were limited and varied among patients, especially in re-subluxated hips.

## Conclusions

PAO combined with or without FO (DRO or VDRO) is effective in treating subluxated hips in children and young adult with CP. Sufficient correction of the acetabular fragment is only possible with complete disconnection of the acetabular fragment. The cuts must be completed with instruments rather than fracturing the final bridges. Solid fixation of acetabular fragment with more screws is mandatory for soft bone.

## Data Availability

The datasets used and/or analyzed during the current study are available from the corresponding author on reasonable request.
